# Developing and Evaluating a Bundled Digital Tool to Improve Complex Care and Self-Management of Patients With Inflammatory Bowel Disease: Protocol for a Hybrid Effectiveness-Implementation Study

**DOI:** 10.2196/65659

**Published:** 2025-08-01

**Authors:** Kaitlyn Delaney Chappell, Melissa Fox, Thomas Scott Armstrong, Lekan Ajibulu, Cynthia H Seow, Aldo Montano-Loza, Karen I Kroeker, Gilaad G Kaplan, Kerri Novak, Christopher Ma, Richard Ingram, Frank Hoentjen, Brendan Halloran, Farhad Peerani, Dina Kao, Karen Wong

**Affiliations:** 1 Division of Gastroenterology, Department of Medicine, University of Alberta, 130 University Campus NW, Edmonton, AB, T6G2X8, Canada, 1 7804928691 ext 6; 2 Division of Gastroenterology & Hepatology, Department of Medicine, University of Calgary, Calgary, AB, Canada; 3 Department of Community Health Sciences, University of Calgary, Calgary, AB, Canada

**Keywords:** digital health, self-management, electronic medical records, quality of life, diet, mental health, inflammatory bowel disease, social determinants of health, implementation

## Abstract

**Background:**

Individuals with inflammatory bowel disease (IBD) require comprehensive care to address the physical and psychosocial burden of their disease. The demand for IBD care often exceeds availability, resulting in delayed access and suboptimal management. As a result, patients with IBD are required to self-manage significant aspects of their disease between appointments with their medical team. Digital self-management tools may help address this gap by empowering patients to be more engaged in managing their disease, potentially improving outcomes and reducing the strain on the health care system.

**Objective:**

This study aimed to design, implement, and evaluate a bundled digital health tool, MyIBDToolkit, with the overarching goal of improving the quality of care and self-management for patients with IBD in Alberta, Canada.

**Methods:**

A bundled digital health tool, MyIBDToolkit, will be integrated into our provincial electronic health record system to ensure broad accessibility and continuity of care. We will use a type 2 hybrid effectiveness-implementation design to evaluate both the clinical impact and real-world integration of the toolkit. We will assess effectiveness through changes in key outcomes such as health care utilization (eg, emergency visits, hospitalizations), disease burden on patients (eg, quality of life, symptom control), and burden on the health care system. These outcomes will be measured using comprehensive health care administrative data. A dual-comparison approach will be used: a within-subject comparison of health care utilization and disease burden before and after implementation of the MyIBDToolkit, and a between-group comparison of outcomes among toolkit users versus nonusers. To evaluate implementation success, we will examine reach (ie, number of patients and providers using the tool), fidelity to the planned timeline, sustained use over time, and factors influencing adoption and maintenance. Our goal is to reach 10,000 patients across Alberta, Canada, within three years.

**Results:**

We received funding for this project in January 2023. In preparation for the pilot launch, we have identified key stakeholders, including patients, health care providers and, administrators, and developed strategies to assess their readiness for MyIBDToolkit. We are also collecting mixed-methods data from patients to explore potential barriers and facilitators to using MyIBDToolkit. The first phase of MyIBDToolkit was launched in October 2024.

**Conclusions:**

MyIBDToolkit represents a scalable and patient-centered approach to the self-management of IBD care. By empowering patients to self-manage their disease between health care visits, we aim to reduce the burden of IBD on patients, providers, and the health care system. By evaluating the effectiveness and the implementation of the MyIBDToolkit, we aim to generate actionable and sustainable improvements to IBD care in Alberta.

## Introduction

Inflammatory bowel diseases (IBDs) are chronic illnesses of the gastrointestinal tract that include ulcerative colitis and Crohn’s disease. IBD is characterized by inflammation of the digestive tract, causing affected individuals to experience remitting and recurring symptoms such as diarrhea, pain, fatigue, and weight loss [1]. The occurrence of these physical symptoms can be challenging to predict, even with optimal medication management [2]. The unpredictability of the disease, combined with the effects of the physical symptoms, can cause IBD to negatively impact additional aspects of a person’s life, including their ability to work, socialize, and engage in leisure [3]. Consequently, this can contribute to poor mental health and quality of life [1,4], highlighting that the effects of IBD extend beyond a patient’s physical well-being.

The interplay between mental health, nutrition, and gastrointestinal health [5,6] highlights the importance of adopting a multipronged approach to the treatment of IBD, that considers the physical and mental effects of the disease [7,8]. While a multifaceted approach to the treatment of IBD is encouraged by most international treatment guidelines [9,10], adherence to multifaceted treatment by health care providers (HCPs) remains low [11], for a variety of reasons, including a perceived lack of time during appointments to discuss factors outside of physical symptoms and medications [12]. This low adherence to a widely accepted approach for treatment emphasizes the need for solutions to help close this gap [13].

Currently, the management of many aspects of IBD, including mental health and diet, falls on the patients, requiring them to self-manage parts of their care [14]. This self-management of the several elements of IBD can be overwhelming and difficult for individuals, and can sometimes result in poor symptom control [15]. A possible solution to improve the self-management of IBD is to leverage the data collection potential of electronic medical records (EMRs), which can allow patients to log and monitor their symptoms between appointments with their HCP [16]. An advantage of EMR use stems from the fact that their HCP can view this patient-entered information, facilitating timely access before the appointment [16,17]. Previously, several studies demonstrated that leveraging EMRs for the care of patients with IBD can improve disease management and contribute to more personalized models of treatment [17,18]. EMRs can be implemented on a large scale and help standardize the quality of IBD treatment while still offering individualized medical aid for those suffering from chronic illness [19].

Starting in 2019, the provincial health care governing body, Alberta Health Services (AHS), began rolling out a provincial EMR system, ConnectCare (Epic Systems). ConnectCare (CC) is a provincially mandated EMR system that allows physicians to access and update patient information across the province. Currently, most major care centers in Alberta have access to CC, with the remaining health care sites being granted access in November 2024. Our study aims to develop and implement a series of health tools into the existing CC infrastructure, thereby standardizing and improving the quality of IBD care in the province.

## Methods

### Ethical Considerations

This study was approved by the University of Alberta Research Ethics Board (Pro00139915). A waiver of consent was approved for the extraction of secondary health data and no compensation will be provided. Participants will provide written informed consent before participating in any of the evaluation surveys. Survey data will be deidentified.

### Objectives and Setting

MyIBDToolkit is a bundled digital tool consisting of patient- and HCP-targeted tools built into CC.

A team of gastroenterologists from sites across Alberta, Canada, were originally assembled to outline what elements would ideally be included in a self-management tool for patients with IBD. Together, they highlighted components of care that may be important for patients to track and the type of additional resources that could help them manage these aspects of their care. Following this, a patient advisory council was assembled to help guide the development of MyIBDToolkit and ensure that it addressed the needs of the patient population. The patient advisory council and the gastroenterology health care provider advisory team co-designed the patient-targeted tools to facilitate patient access to symptom-tracking support, diet education, and mental health resources. Table 1 details all the patient-facing tools that will be integrated into the MyIBDToolkit.

The HCP-targeted tools are designed to standardize evidence-based care and to allow for co-management with other HCPs, such as family physicians and internists, throughout the province. These HCP tools contain embedded decision-support based on the most recent IBD treatment and management guidelines. These tools will also standardize how information about a patient’s disease is entered and collected into CC. Standardizing data entry into a patient’s chart will allow for improved visualization of disease history and facilitate data extraction for future research studies. Table 2 details all the provider-facing tools that will be integrated into MyIBDToolkit.

**Table 1.** Summary of patient-facing tools included as a part of MyIBDToolkit.

Purpose of tools	Details of the tools
Patient-Entered questionnaires and flowsheets	
Symptom tracking and self-management	Stool diary Side effects Missed school/work days Activity indices – Harvey Bradshaw Index [20], partial Mayo Score [21] Flare questionnaire Patient history questionnaire
Quality of life	Health-related quality of life questionnaire
Social determinants of health	Depression screening and inventory using the Patient Health Questionnaire-2 [22] and 9 [23] Anxiety screening and inventory using the Generalized Anxiety Questionnaire-2 and 7 [24]
Mini self-management pathways	
Mental health pathway	Screening tools for depression and anxiety Identify those at risk and referral path to mental health professionals Recommended self-management tools (existing apps/programs, including some local ones vetted for some evidence to support use in IBD^a^ and free to patients)
Diet / nutrition pathway	Screening tools using the Canadian Nutrition Screening Tool [25] and symptoms assessment Locally developed nutrition recommendations and associated resources (developed in collaboration with local IBD outpatients)
AHS^b^ IBD webpage	
“One-stop shop” for patients	Links to IBD information resource Links to credible education resources such as Crohn’s and Colitis Canada, BadGut, and Canadian Digestive Health Foundation Links to self-management pathways Links to ConnectCare patient portal onboarding videos
Implementation outcomes	
Evaluate implementation	Patient satisfaction, usability questionnaires

a IBD: inflammatory bowel disease.

b AHS: Alberta Health Services.

**Table 2.** Summary of provider-facing tools included as a part of MyIBDToolkit.

Purpose of tool	Details of tool
Standardization of care and shared care	
Documentation	IBD^a^ Overview, Pre-testing, Follow Up Smartforms IBD Navigator – “one-stop shop” for all resources/management related to IBD care
Management (as per Clinical Care pathways)	Smartsets, preference lists Decision support Synopsis
Quality of care	IBD Reporting Dashboard Tableau IBD page
Implementation outcomes	
Satisfaction and usability	Provider satisfaction, usability questionnaires

a IBD: inflammatory bowel disease.

### Study Design

The Reach, Effectiveness, Adoption, Implementation, Maintenance (RE-AIM) Planning and Evaluation Framework guided the design of this study [26,27]. The five dimensions assessed by this framework are reach, effectiveness, adoption, implementation, and maintenance.

**Figure 1.**
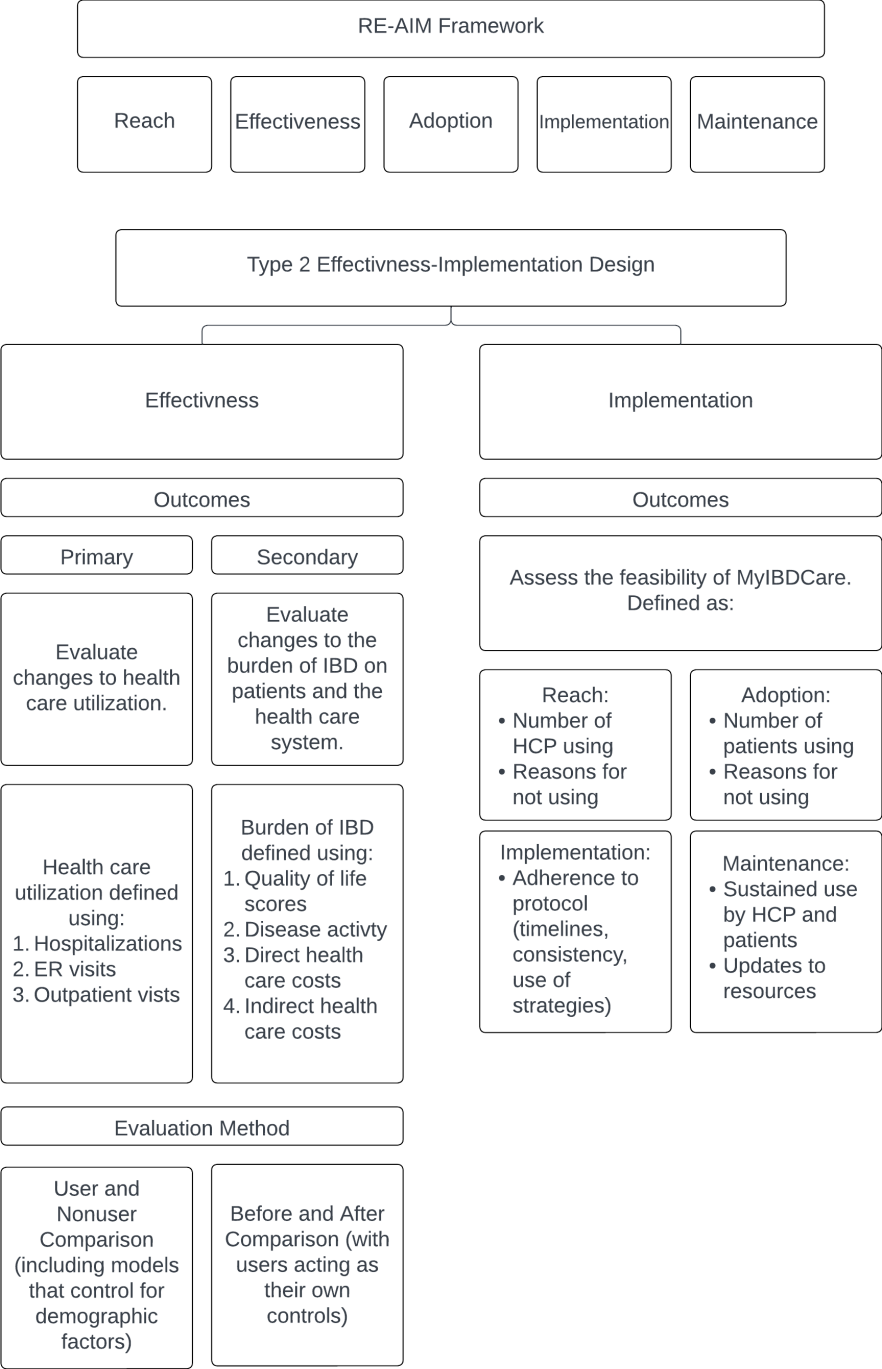
An overview of the type 2 hybrid effectiveness-implementation design to be used. ER: emergency room; HCP: health care provider; IBD: inflammatory board disease; RE-AIM: Reach, Effectiveness, Adoption, Implementation, Maintenance

To evaluate MyIBDToolkit in these five domains, this study uses a type 2 hybrid effectiveness-implementation design, with the effectiveness aim being to determine the clinical effectiveness of the MyIBDToolkit and the implementation aim being to assess the feasibility of the implementation strategy. We will evaluate the effectiveness and feasibility at two-time points: preimplementation and postimplementation, with postimplementation evaluations occurring at one and three years. A visual representation of the trial design is available in Figure 1.

### Participants

MyIBDToolkit will be available to HCPs and patients with IBD with access to CC across Alberta through acute, inpatient, or outpatient care. Providers who do not use CC for outpatient clinical work will be guided to the AHS Webpage for alternative access to the digital tools. Patients will be able to access MyIBDToolkit through their IBD HCP, indicating access to the tool is limited to individuals with a confirmed IBD diagnosis.

We will evaluate the effectiveness and implementation of MyIBDToolkit in six major cities across Alberta: Edmonton, Grande Prairie, Red Deer, Calgary, Medicine Hat, and Lethbridge. We have engaged IBD providers from academic and community sites within each city and will provide resources to them, explaining how to use all the tools within the MyIBDToolkit.

The patients of these IBD providers will be eligible for inclusion in the trial. Strategies to reach patients include providing their HCP with materials, posters, promotional material, and patient handouts to direct them to the AHS Webpage, which explains how to use MyIBDToolkit.

A maintenance of certification program will be created to incentivize HCP engagement and allow them to receive credits toward continuing professional development.

All included HCPs and their patients will be given contact information for a MyIBDToolkit support email. The research team will manage this email, which will help HCPs and their patients work through questions or issues they may have while accessing any of the tools.

### Effectiveness

Effectiveness will be evaluated using a controlled before-and-after study design. Our primary effectiveness outcome is to assess the reduction in patient health care utilization, including hospitalizations, emergency room visits, and outpatient visits.

Our secondary effectiveness outcomes will be to evaluate changes to the burden of IBD on patients and the health care system using quality of life scores, disease activity, maintenance of remission, direct health care costs, and indirect health care costs. A detailed summary of all effectiveness variables that will be extracted for analysis is available in Table 3.

**Table 3.** Effectiveness outcomes to be extracted for analysis.

Outcome	Definition
Number of patient visits to gastroenterology clinics	Follow-up appointments with gastroenterology providers, with the primary appointment reason being IBD^a^
Number of emergency department visits for IBD or IBD-related complications	Emergency department visits with the primary admission or discharge diagnosis noted as IBD or a complication of IBD
Number and length of hospitalizations for IBD or IBD-related complications	Acute hospitalizations and their length with the primary admission or discharge diagnosis noted as IBD or an acute complication of IBD
Number of IBD surgeries for IBD or IBD-related complications.	Surgical procedures with the primary admission or discharge diagnosis noted as IBD or a complication of IBD
Number of patients with IBD prescribed advanced therapy	Biologic therapies and small molecule therapies dispensed for patients with an IBD diagnosis
Cost of advanced therapy for Alberta Health Services	Cost of biologic and small molecule therapies prescribed and dispensed while IBD patient is admitted to hospital
Cost of advanced therapy for patients	Cost of biologic and small molecule therapies dispensed at community pharmacies
Loss of productivity associated with IBD	1. Cost of lost productivity associated with the days patients with IBD spend in emergency departments, in hospital, or in surgery. 2. Work productivity questionnaire
Wait time for first IBD appointment	Number of days between referral for a suspected case of IBD and first appointment with a gastroenterologist
Quality of life scores	The proportion of patients with high, moderate, and low quality of life scores
Disease activity scores	The proportion of patients with active and inactive disease based on Harvey Bradshaw Index and Partial Mayo Score scores
Mental health scores	The proportion of patients with high, moderate, and low mental health scores
Malnutrition scores	The proportion of patients at risk of malnutrition

a IBD: inflammatory bowel disease.

### Statistical Analysis

Relevant IBD records will be extracted from provincial health administrative databases. Historic data on healthcare utilization, from the year before the introduction of MyIBDToolkit, will be used to compare to the rate of utilization in the second year after implementation.

We will evaluate the effectiveness outcomes using two different models:

1. Before-and-after analysis: This will be done by using MyIBDToolkit users as their own controls. We will compare the outcomes of MyIBDToolkit users before the launch of MyIBDToolkit to the outcomes of MyIBDToolkit users after the launch.
2. Users and nonusers: This will be done by comparing the outcomes of patients using MyIBDToolkit to patients not using the tool following the launch of MyIBDToolkit.

To assess the clinical effectiveness of MyIBDToolkit, the interrupted time-series analysis will follow a linear regression model in tandem with standard time-series methods to assess autocorrelation; these tests will include the Durban Watson test.

We based our sample size calculations on the effectiveness outcomes and results from a similar study by De Jong et al [19]. For our sample size calculations, we performed mathematical simulations that accounted for varying panel sizes, rates of visits and admissions for each physician, and rates of uptake by patients within physician panels. The statistical power of the tests was very robust despite variations. In the simulations with our most extreme conditions, (including an average 50% uptake by patients), a sample size of 15 physicians and 4400 patients provided over 90% power to detect the effect on hospital admissions.

### Implementation

Implementation outcomes will be assessed using the RE-AIM framework, and the focus will be on the reach, adoption, and maintenance of MyIBDToolkit as well as the implementation of it.

The outcomes we will be extracting to evaluate implementation include the proportion of patients and providers using MyIBDToolkit, number of MyIBDToolkit tools being used by patients and providers, and the proportion of providers documenting and adhering to IBD quality indicators. These outcomes will be assessed at different time points (ie, yearly) to gain an understanding of the long-term effects of the intervention.

The implementation of MyIBDToolkit will be assessed using both qualitative and quantitative methods, including structured surveys and interviews as well as administrative data. This hybrid approach will allow us to measure reach, adoption, patterns of use, barriers and facilitators, characteristics of users and nonusers, adaptation, and sustainability of use across Alberta. To analyze the data collected, we will use individual-level data in mixed-effects negative binomial regression models that include random patient intercepts and effects nested within physicians, adjusted for age, sex, disease classification, and region.

The duration of the study is 3 years; however, follow-up evaluation will extend to 5 years. Our study will be developed and subsequently launched in a series of phases.

#### Phase 1: Key Stakeholder Engagement

In this stage, we will engage key stakeholders (eg, operations, patient partners, clinicians, nurses, and Digestive Health Strategic Clinical Network (DHSCN)) in the co-development of MyIBDToolkit, which will be crucial, as it will greatly impact uptake and successful implementation. A mixed-method research study will be designed to evaluate the barriers, facilitators, and contextual influences related to using digital resources. This sub-study will be co-designed and co-led by the patient advisory board and will consist of 5‐6 patients or caretakers of varying backgrounds and demographics.

During this phase, research staff will work together to compile and design resources that will be included in MyIBDToolkit.

#### Phase 2: Preparing for Implementation

Clinicians will be approached to participate in a phased roll-out plan in line with CC waves (as HCPs must be CC-trained). At least one clinician from each of the six communities with a large IBD practice has agreed to participate. Additional clinicians with interests in IBD will be approached later by the study leads. MyIBDToolkit will be demonstrated to HCPs (clinicians, nurses, and medical office assistants) through in-person, virtual sessions or videos. Similarly, the study team will demonstrate the patient portal through videos to educate patient participants.

#### Phase 3: Pilot Implementation

MyIBDToolkit will be piloted for 6 months at one site in the first launch city (Edmonton) before expanding to the remainder of the sites in the city. The pilot phase will allow us to identify and resolve any technical or recruitment challenges.

#### Phase 4: Feedback and Rolling Implementation

MyIBDToolkit will be rolled out to the remaining cities after the pilot phase. The phased roll-out will be continued with 1‐2 new cities added every six months. A phased roll-out is necessary to enable the collection and analysis of implementation measurements to help inform and refine the next iteration. The research team will set up mechanisms for timely technology support and feedback. Education videos will be housed on the AHS collection page during and beyond the funding period for HCPs to share with new patients (facilitated by DHSCN).

#### Phase 5: Continuing Improvement and Maintenance

MyIBDToolkit content and the implementation plan will be adapted based on implementation measurement tools and feedback. Technology support and ongoing updates will be used to ensure the accuracy of the content. The postevaluation survey is available in Multimedia Appendix 1.

## Results

We received funding for MyIBDToolkit in January 2023 from Alberta Innovates. The tools that make up MyIBDToolkit are currently in development. The HCP-facing tools have been built into CC and are awaiting final approval from the provincial health care administration. The patient-facing tools have been synthesized, and some original tools to address diet and nutrition are in development.

**Figure 2.**
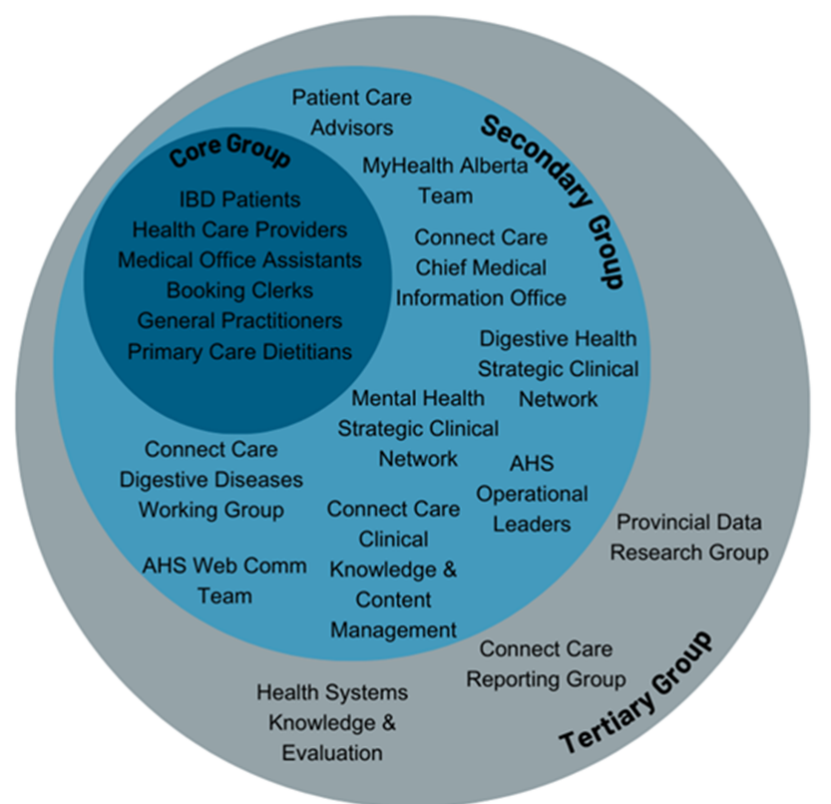
Stakeholders involved in the development and implementation of MyIBDToolkit. AHS: Alberta Health Services; IBD: inflammatory bowel disease.

We have identified all the relevant stakeholders that may be affected by the implementation of MyIBDToolkit. We have divided stakeholders into three groups: core, secondary, and tertiary. The core group represents all the impacted groups that require a practice change or awareness of the MyIBDToolkit intervention. The secondary group outlines all the impacted groups involved in the intervention design and supporting the implementation efforts. The tertiary group represents impacted groups that are involved in the evaluation of the intervention. Figure 2 includes a breakdown of the stakeholders belonging to each group.

As a part of our preimplementation plan, we have engaged patients with IBD from across the province in a mixed methods study. This mixed methods study includes a survey and semistructured interviews and aims to identify the barriers and facilitators to using digital health tools such as MyIBDToolkit. This study took place from May-August 2024 and the findings have been published [28].

As of July 2025, we have begun enrolling patients to evaluate the toolkit.

As a part of our preimplementation plan, we have engaged patients with IBD from across the province in a mixed methods study. This mixed methods study includes a survey and semistructured interviews and aims to identify the barriers and facilitators to using digital health tools such as MyIBDToolkit. This study took place from May-August 2024 and the findings have been published [28].

## Discussion

### Potential Impact

According to a recent study, administrative data suggest that the prevalence of IBD in Alberta in 2014 was 709 per 100,000 persons. Prediction models suggest that the prevalence of IBD in Alberta will continue to rise, with the rate expected to reach 1036 per 100,000 persons by 2035 [29] .

Based on the number of patients with IBD seen by HCPs at the sites participating in our study, we aim to reach 2000 patients with IBD in year 1, 6000 in year 2, and 10,000 in year 3 and engage them in using MyIBDToolkit. Using 10,000 patients as our anticipated study population, we estimate the potential effect of MyIBDToolkit by assuming similar effects to those of a similar bundled digital tool explored in a study by De Jong et al [19] Implementing their tool resulted in an adjusted estimated intervention effect of −0.05 hospital admission per patient and −0.79 outpatient visits per patient over the 12-month study period.

According to provincial administrative health data, the average costs for IBD care in Alberta in 2019 were CAD $9191.14 per inpatient admission and CAD $295.74 per outpatient or ER visit. If the intervention effects are similar to those in the study by De Jong et al [19] study, the reduction in hospital admissions and outpatient visits in our sample population could result in approximately CAD $1.3 million in savings for the health care system in the first year, approximately CAD $4.2 million in the second year, and approximately $6.9 million in the third year.

If MyIBDToolkit is scaled up to the entire province, the reductions in health care utilization may translate into savings of approximately CAD $35 million annually for our provincial health care system, and significant increases in inpatient and outpatient capacity. Although this estimate assumes 100% provider and patient uptake, it does not account for the expected increase in prevalence of IBD over time. these two sources of error are expected to compensate for each other.

### Limitations

Our proposed study is not without limitations. One of the major limitations of our study is the use of administrative databases as primary data source. Health care administrative records can be time-saving and cost-effective for collecting large amounts of data; however, they are limited by the search parameters used to filter the records and the accuracy of the records themselves. This study is also limited as our pilot launch is limited to a single site. Piloting MyIBDToolkit at a single site is advantageous from a logistical point of view; however, it does not allow us to receive feedback from HCPs working at rural sites or HCPs with little experience using CC.

The ability of HCPs and their patients to use MyIBDToolkit is limited by their access to and willingness to use the existing EMR system. By November 2024, CC will be available to all HCPs across Alberta; however, its use is not mandated outside tertiary and acute care centers. This limitation means that some primary care providers or external allied health professionals who support the health maintenance of patients with IBD may not have access to MyIBDToolkit. We have tried to limit this drawback by offering as many of the components of MyIBDToolkit on an external webpage; however, converting all of the resources to print was not possible. AHS continues to advertise CC, and with time, we hope that with more awareness will encourage more primary care providers to join CC and gain access to MyIBDToolkit.

## Supplementary material

10.2196/65659 Multimedia Appendix 1 Post-implementation patient satisfaction questionnaire to be administered following the launch of MyIBDToolkit.

10.2196/65659 Peer-review report 1 Peer-Review Report from Alberta Innovates Grant Committee (Alberta Health Services, Canada).

## Abbreviations

AHS: Alberta Health Services
CC: ConnectCare
DHSCN: Digestive Health Strategic Clinical Network
EMR: electronic medical record
HCP: health care provider
IBD: inflammatory bowel disease
RE-AIM: Reach, Effectiveness, Adoption, Implementation, Maintenance
