# Supplementary material for: Developing and Evaluating a Bundled Digital Tool to Improve Complex Care and Self-Management of Patients With Inflammatory Bowel Disease: Protocol for a Hybrid Effectiveness-Implementation Study
Source: JMIR Res Protoc. 2025 Aug 1;14:e65659. doi: 10.2196/65659 (PMC12360832; doi:10.2196/65659)
Supplement: Multimedia Appendix 1 [file resprot-v14-e65659-s001.docx]

PATIENT SATISFACTION WITH DIGITAL HEALTH TOOLS (MyIBDToolkit) TO IMPROVE INFLAMMATORY BOWEL DISEASE MANAGEMENT

Study ID number: Pro00133852

This questionnaire asks you about a set of digital health tools referred to as “MyIBDToolkit”. These tools were developed in partnership by Alberta Health Services and Alberta Innovates and are available on MyChart (formerly known as MyAHSConnect), the Alberta Health Services website, and the MyHealthAlberta website.

By participating in this study, you will help us gain a better understanding of your satisfaction with the tools available in MyIBDToolkit and identify any remaining barriers and challenges you face when managing your Inflammatory Bowel Disease (IBD).

Your answers are confidential and anonymous. Your responses will not be traced back to you. Your participation in this study will not affect any current or future care.

Participation in this survey is voluntary. We encourage you to answer only the questions you feel comfortable answering.

Do you agree to proceed with the survey?

Yes -> (survey begins)

No -> thank you for your time (ends survey)

# Questionnaire

## General Use of Digital Tools (please consider ALL digital tools you have used)

1. Do you currently, or have you in the past, used digital health tools to help you manage your IBD?
2. Yes, I currently use digital health tools for this purpose
3. Yes, I used digital health tools in the past but do not anymore
4. No, I have never used digital health tools for this purpose
5. Not sure
6. How many digital health tools are you currently using to help manage your IBD?
7. 1-2 digital health tools
8. 3-5 digital health tools
9. 5-9 digital health tools
10. 10+ digital health tools
11. How many digital health tools have you tried to help manage your IBD? This includes tools you currently use and tools you tried in the past but stopped using.
12. 1-2 digital health tools
13. 3-5 digital health tools
14. 5-9 digital health tools
15. 10+ digital health tools
16. Have you accessed your medical chart through MyChart (formerly known as MyAHSConnect)?
    1. Yes
    2. No
    3. Unsure
17. What features do you use in MyChart?

|  | I Frequently Use | I Occasionally Use | I Do Not Use | Not Sure |
| --- | --- | --- | --- | --- |
| To view test results |  |  |  |  |
| To view messages you’ve received from or sent to your healthcare team |  |  |  |  |
| To send a message to ask your healthcare team for medical advice |  |  |  |  |
| To view letters your healthcare provider has shared with you |  |  |  |  |
| To view details of your past appointments or hospital stays (After Visit Summary) |  |  |  |  |
| To send a request for an appointment, view details about future appointments or cancel appointments |  |  |  |  |
| To review your list of medications |  |  |  |  |
| To review and record your personal and family medical history |  |  |  |  |
| To respond to questionnaires sent from your healthcare provider |  |  |  |  |

1. For what purposes have you used digital health tools (currently or in the past)? (check all that apply)

- **Scheduling and Appointments:** to schedule an appointment for your IBD, to keep track of appointments, and to receive reminders about your appointments
- **Symptom Tracking:** to keep track of physical symptoms related to your IBD, and to keep track of your general health
- **Diet Tracking:** to keep track of what you are eating, to keep track of foods and how they make you feel
- **Diet Education:** to learn about what foods you should eat or avoid with IBD
- **Mental Health:** to help you manage your mental health / wellness
- **Medication Tracking:** to keep track of the medications you are taking, to remind you when to take your medications
- **Attending appointments:** to join an appointment for your IBD via video-conferencing (Zoom, Microsoft Teams, etc.)
- **Accessing Personal Medical Information:** to view your electronic medical records (your chart), to view a history of your lab results, to view a history of your tests/procedures, to view a history of your medications
- **Education:** to learn more about your IBD, to learn more about a procedure, to learn more about a medication
- **Communication:** to communicate with your healthcare providers
- **Social Support:** to connect with other people who have IBD
- Other: _______

## Satisfaction with MyIBDToolkit Digital Tools Please note that these tools can be accessed through MyChart (formerly MyAHSConnect), the Alberta Health Services website, and the MyHealthAlberta website.

1. Overall, how helpful or unhelpful do you find the MyIBDToolkit digital health tools available for your IBD?
2. Very helpful
3. Helpful
4. Neither helpful nor unhelpful
5. Unhelpful
6. Very unhelpful
7. How satisfied are you with the MyIBDToolkit digital health tools listed below?

|  | Very Useful | Moderately Useful | Slightly Useful | Not Useful | I Do Not Use | Not Sure |
| --- | --- | --- | --- | --- | --- | --- |
| **MyChart** | | | | | | |
| IBD Stool Chart |  |  |  |  |  |  |
| Missed school/work days |  |  |  |  |  |  |
| Symptom questionnaire |  |  |  |  |  |  |
| Patient history questionnaire |  |  |  |  |  |  |
| Quality of life questionnaire |  |  |  |  |  |  |
| Depression screening |  |  |  |  |  |  |
| Anxiety screening |  |  |  |  |  |  |
| **Alberta Health Services IBD Webpage (www.ahs.ca/IBD)** | | | | | | |
| My IBD Diet: Eating to Lower Inflammation Handout |  |  |  |  |  |  |
| My IBD Diet Plate Handout |  |  |  |  |  |  |
| Alberta Health Services IBD Webpage (www.ahs.ca/IBD) |  |  |  |  |  |  |
| Your Pathway for Managing Anxiety and Depression with Inflammatory Bowel Disease (IBD) |  |  |  |  |  |  |
| Your Nutrition Pathway for Inflammatory Bowel Disease (IBD) |  |  |  |  |  |  |

## Barriers and Facilitators

1. Do any of the following barriers prevent you from using digital health tools? (check all that apply)

- **Lack of Access:** Not having regular access to devices or stable internet.
- **Economic Constraints:** The cost of devices, software, or internet service is a barrier.
- **Skill Limitations:** Lacking the skills or knowledge to use digital health tools effectively.
- **Disability Barriers:** Disabilities make it challenging to use digital health tools.
- **Language Limitations:** Language barriers hinder the use of digital health tools (e.g., non-native language interfaces).
- **Technological Complexity:** Digital health tools are too complex or overwhelming.
- **Privacy and Security Concerns:** Concerts about privacy and security risks in using digital health tools.
- **Awareness of Resources:** Being unaware of the digital health tools available.
- **Lack of Interest/Motivation:** Having little interest or motivation using or learning about digital health tools.
- **Lack of Usefulness**: The information on digital health tools is not helpful or useful.
- **Familiarity:** Being more familiar with print or paper-based health tools and not wanting to switch to digital.
- Other; please specify _____________________________________________

## Demographics

1. What is your IBD diagnosis?

- Crohn's disease
- Ulcerative colitis
- Indeterminate colitis
- Other; please specify ______________________________________________

1. What is your age?

_____

1. At what age were you diagnosed with IBD?

_____

1. What type of community do you live in?

- Urban
- Suburban
- Rural

1. How do you describe your gender identity (check all that apply)?

- Gender-fluid
- Man
- Non-binary
- Trans man
- Trans women
- Two-spirit
- Women
- I don’t identify with any options provided
  - I identify as _______
- Prefer not to disclose

1. Which of the following ethnic categories best describes you? Select all that apply.
   - Black (African, African Canadian, Afro-Caribbean descent)
   - East Asian (Chinese, Japanese, Korean, Taiwanese descent)
   - Indigenous (First Nations, Inuk/Inuit, Métis descent)
   - Latin American (Hispanic or Latin American descent)
   - Middle Eastern (Arab, Persian, West Asian descent; e.g., Afghan, Egyptian, Iranian, Kurdish, Lebanese, Turkish)
   - South Asian (South Asian descent; e.g., Bangladeshi, Indian, Indo-Caribbean, Pakistani, Sri Lankan)
   - Southeast Asian (Cambodia, Filipino, Indonesian, Thai, Vietnamese, or other Southeast Asian descent)
   - White (European descent)
   - Other__________
   - Do not know
   - Prefer not to disclose
2. What is your employment status?

- Employed full-time
- Employed part-time
- Retired
- On paid leave
- On unpaid leave
- Unemployed
- Student
- Unpaid caregiver (providing unpaid support to a family member or friend with a diminishing physical ability, a debilitating cognitive condition or a chronic life-limiting illness)
- Stay-at-home parent (providing unpaid childcare to children you are a parent or guardian to)
- Other; please specify ______________________________________________
- Prefer not to answer

1. What is the highest level of schooling you have completed?

- Some high school complete
- High school
- Post-secondary certificate or diploma
- Bachelor’s degree (BA, BSc, BEd, etc.)
- Graduate degree (MA, MSc, MPH, PhD, etc.)
- Professional degree (MD, JD, DDS, etc.)
- Other, please specify

**Thank you for your participation! Would you like to participate in an hour-long follow-up qualitative study about digital tools and IBD?**Your participation in an interview will help us better understand how you manage your IBD and improve the digital tools available to people with IBD. Interview participants will be compensated for their time.

If you select YES, you will be asked to provide your phone number. A research team member will follow up with you regarding the interviews.

**o Yes o No**

Please provide your preferred phone number: ______________
