# Supplementary material for: Developing and Evaluating a Bundled Digital Tool to Improve Complex Care and Self-Management of Patients With Inflammatory Bowel Disease: Protocol for a Hybrid Effectiveness-Implementation Study
Source: JMIR Res Protoc. 2025 Aug 1;14:e65659. doi: 10.2196/65659 (PMC12360832; doi:10.2196/65659)
Supplement: Peer-review report 1 [file resprot-v14-e65659-s002.pdf]

Lead: Wong, Karen (University of Alberta)

Application #: 222300176

Project Title: Patient Dashboard (PD-IBD) to Improve Complex Care and Self-management of Inflammatory Bowel Disease (IBD)

Reviewer Comments

Opportunity (Problem & Solution):

- It is not clear why IBD is expected to increase over the coming years.
- Equity is assumed to comprise geographic (in)equity given the urban-rural disparities that are noted.
- The proposal outlines the evidence from other jurisdictions and in Alberta on each distinct element (of the four key elements) but not evidence of the effectiveness of the full bundled solution.
- Consistency in evidence presented – evidence of effectiveness for this patient population.

KM:

- The KM strategy represents co-design through all facets of the work.
- The plan involves routine sharing of results and data with key stakeholders.
- A major element of KM that appears essential for this proposal to success is the engagement and buy-in of providers – the proposal could provide additional detail for provider engagement, in urban and rural regions.

Relevance:

- The alignment with PRIHS objectives is rooted in cost savings. (annual cost savings estimated at $10.6 million - based on reduced hospital admission, outpatient visits, and ED visits)

Implementation Plan:

- Ambitious proposal.
- The intervention includes 4 key elements, each individually tested elsewhere but have yet to come together as a package, which introduces some uncertainty and some innovation to the plan.
- There appears to be significant engagement and developmental work needed, with (acknowledged) uncertainties around physician & patient interest and uptake.
- Success depends on several factors outside the team's control, such as rollout of Connect Care (CC) as planned and approval to implement within CC. The plan is ambitious in that it entails engagement to inform co-development, co-development of the tool itself, implementation, and evaluation – in the three-year time period.
- Consider outlining how partients and providers will be recruited.
- This is exactly how the patient portal should be used – feasible from an IT perspective
- The stated mitigation strategies for clinician barriers include "integration of all tasks and reminders so that they occur at point-of-care as part of the workflow" – this will be necessary but may not be sufficient to confirm provider buy-in and use.

Methodology:

- This study employs a type 2 hybrid effectiveness-implementation design to determine the clinical effectiveness of the PD-IBD with ITS to compare outcomes pre- and post-implementation and users vs non-users.
- To better understand factors related to uptake and success, individual-level data in mixed effects negative-binomial regression models will be used that include random patient intercepts and effects nested within physicians.
- The modelling of economic benefit depends on the successful design and implementation and uptake of the solution.

Team:

- The team is large and impressive.
- It appears that there has been a good amount of engagement to date to explore support for the project.

Work Plan and Budget

- All activities seem to be funded through the PRIHS budget; no other cash or in-kind contributions from project partners are noted.
- The proposal should consider longer-term system costs and how they will be funded to sustain the initiative.

Impact:

- The potential for impact is important and impressive - including benefits to patients, providers and the system.
- The impacts section includes a lengthy list of potential impacts ranging from reduced travel and patient out-of-pocket costs, to reduced workload for administrative staff, and more. It is not clear if and how the full scope of these impacts will be assessed.

Strengthening Recommendations:

- Overall this is an impressive, important and ambitious proposal.
- Consider engaging more patient partners on the team.
- Consider including any evidence that the the proposed solution is of interest to patients and providers – (i.e. that this is user-centred solution)
- Consider including additional detail about the specific solution components and barriers and enablers to implementation.
- Consider consulting with IT to determine strategies to market the tool to patients regarding how patients should use/interface it.
